# Impact of the Gastro-Intestinal Bacterial Microbiome on *Helicobacter*-Associated Diseases

**DOI:** 10.3390/healthcare7010034

**Published:** 2019-02-22

**Authors:** Maxime Pichon, Christophe Burucoa

**Affiliations:** 1 Bacteriology and Infection Control Laboratory, Infectious Agents Department, University Hospital of Poitiers, 86021 Poitiers, France; christophe.burucoa@chu-poitiers.fr; 2 Laboratoire Inflammation, Tissus Épithéliaux et Cytokines, EA 4331, Faculté de Médecine et de Pharmacie, University of Poitiers, 86022 Poitiers, France

**Keywords:** gastrointestinal tract, bacterial infections, stool microbiome, 16S, whole genome sequencing, NGS, *Helicobacter pylori*, gastrointestinal cancer

## Abstract

*Helicobacter pylori* is a bacterium that selectively infects the gastric epithelium of half of the world population. The microbiome, community of microorganisms gained major interest over the last years, due to its modification associated to health and disease states. Even if most of these descriptions have focused on chronic disorders, this review describes the impact of the intestinal bacterial microbiome on host response to *Helicobacter* associated diseases. Microbiome has a direct impact on host cells, major barrier of the gastro-intestinal tract, but also an indirect impact on immune system stimulation, by enhancing or decreasing non-specific or adaptive response. In microbial infections, especially in precancerous lesions induced by *Helicobacter pylori* infection, these modifications could lead to different outcome. Associated to data focusing on the microbiome, transcriptomic analyses of the eukaryote response would lead to a complete understanding of these complex interactions and will allow to characterize innovative biomarkers and personalized therapies.

## 1. Introduction

### 1.1. Burden of Helicobacter-Associated Infectious Diseases

*Helicobacter pylori* (*H. pylori*) is a Gram-negative bacterium that selectively infects the gastric epithelium of half of the world population [1]. The prevalence of *H. pylori* infection is highly variable among various countries and even within countries, reaching up to 70–90% in developing countries and in populations originating from high-prevalence countries [2]. Infection by *H. pylori* is usually acquired during early childhood with an intrafamilial transmission, and in most cases persists unless eliminated by antibiotic treatment [3]. Chronic infection by *H. pylori* leads to local inflammation of the gastric mucosa (gastritis), without clinical symptoms in most infected subjects. Although gastric infection with *H. pylori* induces histological changes in the gastric mucosa in most infected individuals, only a minority develops severe gastroduodenal diseases (ulcers, precancerous and cancerous lesions) [4]. Among these infected subjects, approximately 10% develops peptic ulcer disease, 1 to 3% gastric adenocarcinoma, and less than 0.1% gastric mucosa-associated lymphoid tissue (MALT) lymphoma. *H. pylori* is considered as a type I carcinogen (for the International Agency for Research on Cancer), and gastric cancer is the fifth most common malignancy worldwide [5]. Among these *H. pylori* induced gastric cancer, intestinal-type adenocarcinoma, the most frequent type, is initiated by the transition from normal mucosa to chronic superficial gastritis. Progression through a series of well-defined histological steps from atrophic gastritis is followed by intestinal metaplasia leading to dysplasia and adenocarcinoma [6,7]. Except for the carcinogen role, the role of *H. pylori* infection on idiopathic thrombocytopenic purpura, sideropenic anemia, and vitamin B12 deficiency has been established, with a growing interest in other conditions, such as cardiovascular, neurologic, dermatologic, immunologic, and metabolic diseases [8].

Eradication therapy is recommended for peptic ulcer disease, MALT lymphoma, atrophic gastritis, as for first-degree relatives of gastric cancer patients, unexplained iron deficiency anemia and chronic idiopathic thrombocytopenic purpura. Depending on resistance status of the isolated strain (or local epidemiology), different antibiotic treatment (association mostly) can be proposed, associated with a proton pump inhibitor and/or bismuth salt [9].

In infected patients, *H. pylori* is the dominant bacterial species in the gastric microbiota [10]. The cultivable and spiral shape form of *H. pylori* is present only in the gastric mucosa of the human stomach. Coccoid forms of the bacteria are present in the feces of infected patients, their DNA can be detected by PCR, but this form of the bacteria cannot be grown (see parts thereafter). Coccoid form is considered as viable not-cultivable form of the bacteria by some authors with a role in transmission and as non-viable degenerative forms of dying bacteria [11].

Following evolution of the molecular technologies, new paradigm and characterization were developed, including infection by *H. pylori* in the large field of research of the microbiome.

### 1.2. The Association between Helicobacter-Associated Disease and the Bacterial Microbiome: A Lot to Learn

The microbiome could be defined as the whole microbial community, present in (and on) the body of a defined host [12]. Even if this notion is widely used in studies, the precise definition remains controversial. Considered as a “collection of microbial genome” (genomic definition), the microbiome could also be enlarged to the “whole ecological niche including the colonized habitat” [12]. If most studies focused on its bacterial component, in humans, the microbiome is constituted of more viruses than bacteria and contained also fungi and *Archeae* [13,14].

Studies in general population have focused on the characterization of bacterial microbiome depending on the considered niche, bringing important information on structure and variation over time. It is important to know that the microbiome varies deeply among individuals and that colonization by a certain type of bacteria could confer protection or susceptibility to disease, especially in chronic infection as for infection by *H. pylori* [15].

**Figure 1:**
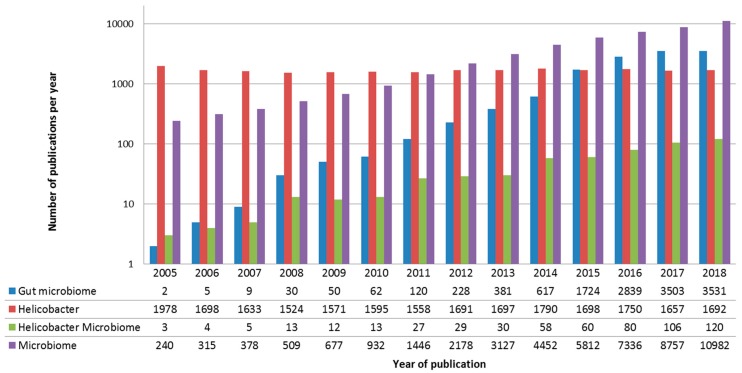
Bibliometric parameters of microbiome and *Helicobacter* studies from 2005 to 2018. This figure represents results per year of four different researches on PubMed (“Microbiome” in purple, “*Helicobacter*” in red, “gut microbiome” in blue and “*Helicobacter* microbiome” in green). If most of these studies on *Helicobacter* remain constant on this period, the number of publications focusing on the microbiome (especially gut microbiome) dramatically increase since democratization of Next-Generation Sequencing (NGS) technologies in 2010.

More than 10,000 articles were associated with the “microbiome” keyword in PubMed in 2018, a major part focusing on intestinal tract microbiome. Moreover, if most of these publications studied the gut microbiome, a very small proportion focused on microbiome disturbance associated to *H. pylori* diseases (Figure 1). Given that studies mainly described role and composition of the bacterial microbiome, especially during *H. pylori*-associated diseases, we will restrain on it in this review.

## 2. Impact of the Microbiome on Intestinal Diseases

Characterization of the composition and variation of the “healthy microbiome” has been performed during a first period. Since then, more recent studies focused on modification during a pathologic state (symptomatic or not). Due to logistic difficulties to sample a patient before a bacterial or viral infection (except during challenge studies), few studies focused on the immediate modification during infectious disease.

Studies have focused on the structural composition of the microbiome, independently to the considered ecological niche. Two level of microbiome could be considered. First, the “core” microbiome, constituted of the bacterial species present in more than 95% of the sampled sites in a niche, is mostly shared between healthy people. This microbiome include bacterial gene and metabolic pathways, implicated in “normal” physiology and varies between patients depending on intrinsic (e.g., age, sex, and social characteristics) and/or extrinsic characteristics (such as alimentation or drug abuse) [16,17]. If microbiome composition deeply varies during the first year of life, bacterial profiles converge quickly since the age of two months to a mature and stable profile. At contrary, the “satellite” microbiome, corresponding to the remaining five other percent, varies frequently, rapidly, and reversibly. These variations are mostly associated with acute diseases (both infectious and non-infectious diseases), pregnancy, aging, or therapeutic use. If this microbiome reflects in detail the present situation of the sampled patient, transversal comparison between studies are difficult, justifying the need for longitudinal studies [18,19,20,21].

To overcome this difficulty, experimental infections on animal models provide some mechanistic information, even if animal microbiome could not be considered as completely as the human one [22]. Moreover, lots of studies have analyzed trans-kingdom interaction within the human microbiome, especially the gut microbiome. For example, bacteria and bacteriophages’ interactions are deeply described in the gastrointestinal tract. For example, bacteriophages could alter the bacterial microbiota by lytic or lysogenic infections of their hosts [18]. By these, bacteriophages bound to the intestinal mucosae are considered to provide immunity to specific pathogens [23].

The intestinal microbiota could impact outcome of viral or bacterial infection through bacteriome–bacteriome or bacteriome–virome interactions. Therefore, to ease the understanding of the subject, this review will be limited to interactions between bacterial microbiota and a single pathogen, *H. pylori*. However, how the whole intestinal microbiota is associated with *H. pylori* infections severity remains still largely unexplored (Table 1).

### 2.1. How to Analyze Bacterial Intestinal Microbiota

Before giving information about the impact of intestinal microbiota disturbance on pathologies, this review will give some information on the analysis itself. Most of the microbiome studies used amplicons sequencing, focusing on 16S rRNA-coding gene. This gene exists in all bacterial reign, containing ten constants (C1 to C10) and nine variable (V1 to V9) regions. These latter are used for taxonomic analyses [28,29]. At first analyzed by Restriction Fragment Length Polymorphisms analyses (RFLP), the emergence of next-generation sequencing (NGS) was responsible for a true revolution in microbial ecology studies (“16S analysis”) [30]. These technologies allow classifying reads into Operative Taxonomic Units (OTU), by using different software such as mother, QIIME2, or UPARSE [31,32,33]. Nevertheless, some studies have demonstrated that this approach, compared to whole genome sequencing, underestimates the bacterial diversity [34].

Moreover, it is important to keep in mind that 16S analysis does not bring information on the viability of the detected bacteria. To analyze this parameter, a shotgun sequencing of bacterial mRNA has to be performed [16]. For the rest of this review, we will distinguish targeted metagenomic (16S analysis approach), shotgun metagenomic (complete sequencing of a sample), and meta-transcriptomic (sequencing of all transcribed gene in a sample), key for the understanding of their impact.

### 2.2. Gut Microbiota Structure and Evolution

Bacterial concentration increases all along the gastrointestinal tract (GIT) with the lowest bacterial concentration in the stomach (10^2^ bacteria per gram content) and the highest concentration in the distal colon (10^12^ bacteria per gram content) [35]. This difference in bacterial concentration could be associated to differences in pH condition, with inhospitable acidity in stomach leading to a severe limitation in bacterial growth or survival.

**Figure 2:**
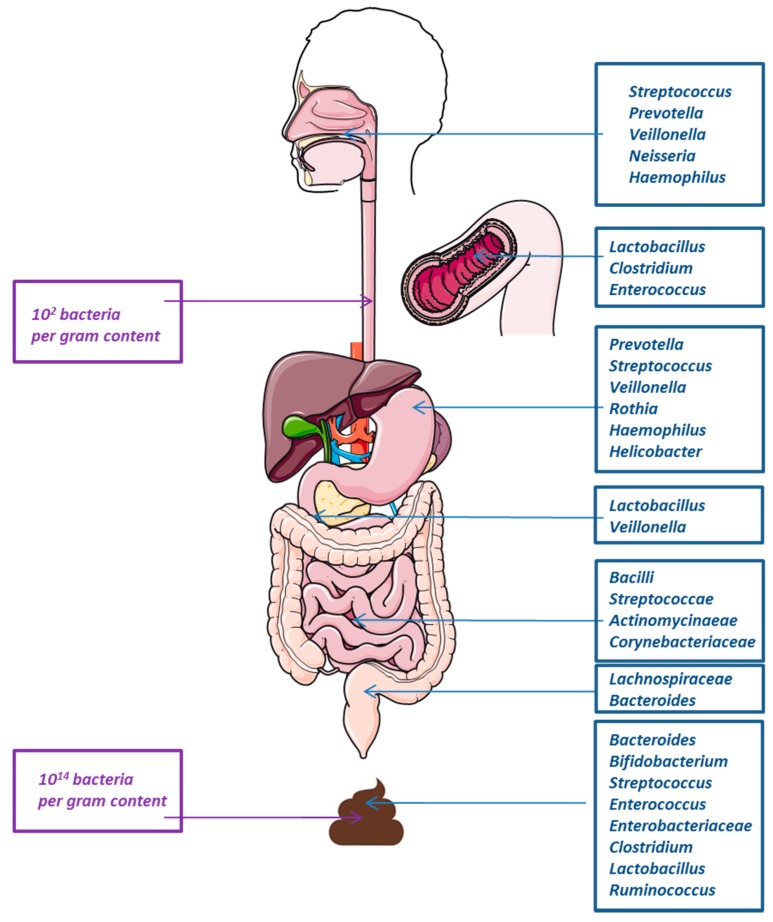
Bacterial microbiome composition and abundance in the gastrointestinal tract. From upper to lower gastrointestinal tract, bacterial microbiome concentration varies between 10^2^ to 10^14^ bacterial per gram of content (left of the figure). This composition varies dramatically between intra epithelial and intra luminal content and depends on the intestinal level (major species indicated at the right of the figure).

Gut microbiome composition varies between patients and some information could be highlighted (Figure 2). If most of the human gut microbiome include anaerobic bacteria (*Bacteroidetes, Firmicutes* or *Proteobacteria*), minor frequency bacteria (<1% in frequency) belong to other phyla (*Actinobacteriae*, *Acidobacteriae*, *Verrumicrobiae*, or *Fusobacteriae*) [36,37]. When focusing on specific level of the GIT, difference could be observed in bacterial composition [35].

A difference between epithelium and intestinal lumen is added to this level-specific variation. This difference could be explained by the production of mucus by the goblets cells, limiting adherence and invasion capability to the “specialized” bacteria. For these bacteria, including *Clostridium*, *Lactobacillus*, or *Enterococcus*, glycosylated proteins (constituting the mucus) are a nutrient tank, used before accessing to the intestinal cells [38]. These mucosa-associated bacteria are less studied than bacteria found in the feces, due to the difficulty to sample biopsies compared to stool samples. Differences have been demonstrated between diversity and compositions of biopsies or stool [35]. Finally, close contact of these bacteria with host intestinal cells may be essential to understand their impact on immune system, justifying the need to study these bacteria [39].

### 2.3. Bacterial Microbiota Direct Interaction with the Intestinal Tract Environment: The Mucosal Interface

Intestinal tract contains cells and a glycoproteic layer, thicker in the distal GIT than in the proximal GIT. This mucus is a highly effective and a specific mechanism of defense against micro-organisms. Certainly, its structure limits penetration of bacteria and destruction of the epithelium from the very important number of bacteria in the GIT. The glycosylation structure, highly conserved between humans, demonstrates its important role in the selection of intestinal microbiome. Indeed, only some rare microorganisms possessing lectins could adhere to mucus layer [38]. If these bacteria could be beneficial (as for *Lactobacillus reuteri*, *plantarum*, or *rhamnosus*), pathogenic ones have also been identified (as *Helicobacter pylori* or *Campylobacter jejuni*) and have shown the capacity to bind blood group antigens present in this mucus layer [38].

Difference in mucine-binding and/or mucine-degrading bacteria (as *Akkermansia muciniphila*), maintaining intestinal integrity, were observed between “healthy” and “inflammatory” GIT. Higher concentration of these bacteria has been found in healthy individuals [40]. Furthermore, germ-free mice models demonstrated that microbiota could be necessary to form the mucus layer, by enhancing the number of goblet cells, the cells responsible for mucine production [36]. It is to note that an important impact on the global immune response has also been shown, as these mice presented altered immune responses with limited cytokine and immunoglobulin concentrations [41]. Only some rare microorganisms possessing lectins could adhere to the mucus layer [38]. If these bacteria could be beneficial (as for *L. reuteri*, *L. plantarum*, or *L. rhamnosus*), pathogenic ones have also been identified (as *H. pylori* or *Campylobacter jejuni*) and have shown the capacity to bind blood group antigens present in this mucus layer [38].

This information highlights that more than just an interaction element with intestinal frontier, microbial communities, constituting the gastrointestinal microbiome represent a true component of this barrier. The intestinal epithelium represents a real interface between intestinal lumen (superficial) and muscular structure (deep). It is mainly constituted of enterocytes and non-epithelial cells, and tight junctions play a dramatic role in this protection [42]. 

During Crohn’s disease, injured regions have been associated with modification of the associated microbiome, with and increased proportion of *Proteobacteriae* (mainly *Escherichia*), and a reduced proportion of *Firmicutes* (including *Lachnospira*, *Faecalibacterium*, and *Blautia*), favoring deep lesions to the endothelial cells [43]. Similarly, dysbiosis and bacterial invasion have been described in the gut of a patient with inflammatory rheumatisms, as in ankylosing spondylitis [44].

Globally, these data highlight the interplay between microbiota and host epithelial cells, an interaction that must be explored deeply to improve understanding of these changing conditions during autoinflammatory or cancerous diseases.

### 2.4. Bacterial Microbiota Interaction with Innate and Adaptive Immune Systems

GIT interacts deeply with immune system, as lymphoid tissue could be localized all over the intestinal tract, leading to maturation of the host defense and to gut homeostasis maintenance. Indeed, this immune system is constantly stimulated by microbial antigens (invasive or not, commensal or not). Paneth cells, deep inside the intestinal crypts, were implicated in the secretion of antimicrobial peptides (such as lectins, lysozyme, or defensins) implicated in bacterial regulation to preserve gut integrity [45].

Intestinal and dendritic cells, present in high numbers in the GIT, could be stimulated by Pattern Recognition Receptors, such as Toll-like receptor-4 (detecting lipopolysaccharide for Gram-negative bacteria or nucleic acids for viruses) or TLR-2 (detecting peptidoglycan or lipoteichoic acids for Gram-positive bacteria). This stimulation leads to the recruitment of immune cells implicated in both native and adaptive systems (T cells, B cells, macrophages) but also to the secretion of enzymes and chemical mediators [46]. Moreover, dendritic cells, present in the mucosal structure of the GIT, capture bacteria and keep them alive to stimulate mesenteric lymph nodes’ plasmocytic cells, with stimulation leading to the secretion of bacteria-specific immunoglobulin A. This adaptive response is implicated in the inhibition of growth and penetration of bacteria, limiting bacterial sepsis [47,48]. Because they are recognized in a similar way to pathogenic bacteria, bacteria constituting a healthy microbiome could modulate inflammatory responses. Some constituents, such as *Bacteroides fragilis*, are responsible for a tolerogenic response (activating regulatory T cells, secreting interleukin-10) [46,49]. Similarly, other commensal GIT bacteria, including *E. coli*, *Enteroccocci*, or *Bacteroides*, induce the maturation of dendritic cells, and provokes the secretion of both pro- and anti-inflammatory cytokines, highlighting their impact on the maturation of the immune system [50].

It has been demonstrated for years that host immunity can inhibit or promote tumor formation. In a mice model, *Fusobacterium nucleatum* enhanced neoplasia development by limiting antitumor T cell-mediated immunity [51]. More than just host immunity, inflammation is a key factor associated to cancer development. It has been shown in inflammatory bowel diseases that gut microbial structure is modified. These modifications are associated with cancer development (mutation of tumor p53 protein, activation of β-catenin, Wnt pathways, cytokines or DNA alteration) [52,53].

Altogether, these data highlight the dramatic impact of bacterial microbiome structure on different aspect of innate and adaptive immune system, especially in a rich and complex microbiome as the intestinal microbiome.

### 2.5. Relationship between Helicobacter pylori and the Gastric Microbiome

If culture-based studies have suggested that stomach is physiologically a sterile niche, recent advances have shown that gastric microbiome is exceedingly rich and complex (10^2^ to 10^4^ colony forming units per gram content). This microbiota, in healthy individuals, is mainly composed by *Veillonella* sp., *Lactobacillus* sp. and *Clostridium* sp. and/or *Propionibacterium*, *Streptococcae*, and *Staphylococcae*, depending on the isolation methods, independently to the anatomical site of sampling [19]. Using 16S analysis, studies have shown that major phyla to compose microbial communities of the stomach were *Proteobacteria*, *Firumicutes*, *Bacteroidetes*, *Actinobacteria*, and *Fusobacteria* [20]. At the genus level *Streptococcae* and *Prevotella* are the most abundant genus in healthy patients [21].

This microbiome could be modified during chronic *Helicobacter pylori* infection, likely due to the change in gastric physiology. It has been observed a reduced gastric acidity, an alteration in innate response and a modification of nutrient availability [54]. If this effect remains incompletely understood, it has already been shown that colonization by *Helicobacter pylori* could modify relative abundance of *Lactobacillus* spp.; *Firmicutes* and *Bacteroidetes* [24,55]. Maldonado-Contreras et al. have shown that chronically infected patients have a higher abundance of *Spirochetes*, *Acidobacteriae*, and *Proteobacteriae*, associated to a decrease of *Actinobacteriae*, *Bacteroidetes*, and *Firmicutes* [10]. At contrary, clearing of *Helicobacter pylori* by antibiotics have shown (by culture method and not high-throughput sequencing) a significant increase of *Enterococci*, *Enterobacteria*, and *Peptostreptococci*. In the same time, anaerobic bacteria (as *Bifidobacteria* or *Clostridia*) were slightly suppressed [25].

During evolution, inflammation results in gastritis. Compared to *Helicobacter*-negative patients, microbiota of these patients shows a decrease of *Proteobacteria* (*Prevotella*) and an increase of *Firmicutes* (*Streptococcus*) [22,56]. It is to note that no difference could be shown between anatomical sites of the stomach in terms of bacterial microbiome. Finally, during gastric transformation to an invasive cancer, bacterial diversity tends to decrease with a major decrease in *Porphyromonas*, *Neisseria*, and *Streptococcus sinensis*, associated to an important increase in *L. coleohomonis* and *Lachnospiraceae.* The genus *Pseudomonas* is one of the most abundant taxa constituting microbiome of gastric cancer [27].

**Figure 3:**
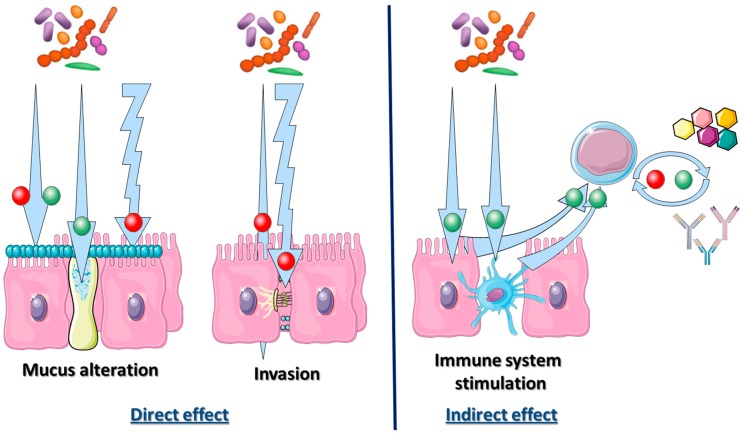
Interaction of the host immune system and bacterial gut microbiome during *Helicobacter pylori* infections. Red and green point in arrows symbolize interactions responsible for severe or mild diseases respectively. Bacterial microbiota could have a direct effect on gastrointestinal tract or an indirect effect on immune system activation. The direct effect of bacterial microbiome could enhance (or decrease) bacterial adhesion into mucus layer. This mucus production could be stimulated by some bacteria, but this layer could be degraded by them. As for destruction of intercellular tight junction, this degradation could enhance bacterial invasion through epithelial mucosa. Finally, bacteria activating TLR could lead to dendritic cells activation as for B or T lymphocytes, and monocytes. This response could be beneficial or detrimental depending on its intensity.

If these results suggest a close relation between *H. pylori* and the remaining stomach microbiome, the role of the gastric microbiome in intragastric colonization of *H. pylori* has not yet completely been understood (even if some effect have already been described; Figure 3). 

Finally, as published studies have already explored the complex interaction between microbiome and immune response to bacterial infection, some authors highlighted that their data could be used in clinical practice to optimize management of *H. pylori*-infected patients, especially when cancerous consequences could occur and be prevented.

## 3. Clinical Use of the Gastro-Intestinal Microbiome Impact during *Helicobacter*-Associated Diseases: State-of-the-Art and Future Perspectives

### 3.1. Could Stool Microbiome be Used to Detect Gastric Infection by Helicobacter pylori?

As described previously, GIT microbiome is modified at each step of the clinical evolution of the gastric inflammation or (pre-)cancerous lesions. Searching for these modified bacteria to quantify them (by molecular biology) in non-invasive sample as stool samples could be an important optimization of the clinical management of infected patients. In a manuscript in preparation, our team has observed modification of the stool microbiome of *Helicobacter*-infected patients, allowing a complete non-invasive diagnosis of *Helicobacter* infection (with stratification depending on the histological state of the gastric disease).

Moreover, *Helicobacter* antigen detection in the stool has been used for years to diagnose *Helicobacter* infection when invasive samples (gastric biopsies for example) [57]. Using ultra-deep sequencing of the stool microbiome, the performance of detection could be impressive and optimize clinical management dramatically. A need correlate bacterial detection/quantification in stool remain to be set up before applying this method to specific cases. This approach could be considered, for now, too expensive, except for specific situation as for patients with risk factor or associated to prognostic determination of *H. pylori*-associated diseases.

### 3.2. Could Characterization of the Stool Microbiome Be Used as a Prognostic Biomarker to Detect Helicobacter-Associated Complications?

As described in the first part of this review, *Helicobacter pylori* presents a dramatic association with carcinogenesis of gastric cancers. Other oral microbiota, also found in the gastric microbiota could be associated with this pathogenic development (as for *F. nucleatum*, *Parvimonas micra* or *Peptostreptococcus stomatis*, *Streptococcus anginosus*, *Dialister pneumosintes*, *Slackia exigua*, *C. colicanis*) [58,59]. This modified gastric microbiome could be used as a predictive biomarker [60].

Present in massive quantity in the gut, it is easily understandable that some bacteria could be associated with cancer development. For example, *Bacteroides fragilis*, secreting multiples toxins, present some precancerous capacities [61,62,63]. Moreover, studies have demonstrated that the modification of the GIT microbiota (in diversity and composition) during or after colon cancer development could be used for identification of cancerous patients [64,65]. Indeed, this pathological state demonstrated for example, a decrease of *Lactobacillus* or *Bifidobacterium* and an increase of *Fusobabacterium*, *Peptostreptococcus*, or *Staphylococci*. In parallel, several studies support a protective role of bacteria, as *C. butyricum, Bifidobacterium*, *L. casei* and *Bacillus subtilis* to inhibit tumor dysbiosis in mice [66,67,68,69].

These different modifications could be, after important technical development, used in routine practice to detect variation associated with severe diseases as cancerous states.

### 3.3. Could Stool Microbiome Characterization be Used to Predict Therapeutic Effect of Anticancer Chemotherapies?

Gastric cancer could be treated using cyclophosphamide and immunotherapies (as anti-PD-1 antibodies). Considering that it has been shown since 2013 that the gut microbiome could modify therapeutic response to the adapted anticancer chemotherapies, its deep impact for carcinogenesis is clear, but could be also considered as impacting cancer therapy efficiency [70]. For example, it has been shown that two bacteria (*E. hirae*, *Barnesiella intestihominis*) have demonstrated the capacity to potentiate the effect of cyclophosphamide. Administration of anti-Gram-positive antibiotics prior or concomitantly to anti-cancerous cure has also shown a detrimental effect on the response to therapeutics [71]. Similarly, abundance of *Akkermansia* is positively correlated with the efficacy of anti-PD-1 antibodies. In front of these data, authors have suggested that the susceptibility to this treatment can be transmitted by transfer of a responder microbiota [72].

Altogether, these data highlight the benefit to clearly identify beneficial and detrimental bacteria in a stool microbiome, to predict therapeutic issue. This approach could be applied in gastric cancer management, to potentially optimize therapies. 

### 3.4. Could Stool Microbiome Characterization Be Used to Modify Response to Therapies?

As a clear suspicion of the implication of a well-adapted microbial structure and repartition in pathophysiological development, lots of studies aimed to identify bacterial that could enhance therapeutics effects. That is the reason of a sudden gain of attention for prebiotics and probiotics.

Prebiotics, mainly constituted of oligosaccharides, could help probiotics to settle and multiple. They could also reduce the concentration of pathogens and toxins, or increase production of energy source for enterocytes [73,74]. Probiotics, defined as “organisms with a potential beneficial effect onto disease development and impact” contain mostly *Lactobacillus* or *Bifidobacterium* sp. Their beneficial effects include production of defensins and/or vitamins and modification in gut permeability [73]. There has been demonstrated in inflammatory bowel diseases (IBD) as for ulcerative colitis or Crohn’s disease [74].

A general consensus exists that IBD is associated with compositional and metabolic changes in the intestinal microbiota [75]. *H. pylori* infection is negatively associated with IBD regardless of ethnicity, age, and *H. pylori* detection methods [76]. Antibiotics used to treat IBD appear to amplify this negative association [76]. Conversely, the use of antibiotics for *H. pylori* eradication therapy (association of two or three antibiotics) could trigger a recurrence or exacerbation of IBD, even if limited published data support this hypothesis [77]. Three cases of Crohn’s diseases exacerbation after *H. pylori* eradication have been already described [78,79]. At contrary, eradication of the bacteria did not significantly change disease activity measures or the presence of gastro-duodenitis in a small cohort study of six patients with quiescent Crohn’s disease [80]. Further studies are needed to reveal the relationship between *H. pylori* eradication and IBD onset or progression.

For sure, after extensive demonstration and clinical studies, use of these innovative molecules/strains will be of interest to optimize therapies, especially therapies targeting chronic inflammatory diseases, as *Helicobacter*-associated diseases.

## 4. Conclusions

Since the first descriptions of the Koch’s paradigm and the description of the impact of *H. pylori* infection, optimization in the management of infected patients benefited of the bacteria deep characterization. If the development of ultra-deep sequencing allowed precise studies of the pathogen genome describing resistance- or virulence-associated mutations, we have highlighted herein that the microbiome could be strongly implicated in the *H. pylori*-associated diseases pathogenicity. Furthermore, the implication of host immunity in the development of cancerous or inflammatory diseases is well described, justifying the study of the cellular response. An integrative approach, including data focusing on the pathogen, the microbiome and the host response, must be considered to fully understand a disease, especially infectious one [81]. After completion of a mechanistic understanding on animal models, microbiome information will open a new field of research for clinical management.

## Figures and Tables

**Table 1:** Modification of gastric bacterial microbiome composition and abundance during *Helicobacter*-associated disease.

Analytical Method	Pathophysiological Context	Modification in Bacterial Abundance	References
Culture method	Healthy patient	*Veillonella* sp., *Lactobacillus* sp., *Clostridium* sp. *Propionibacterium*, *Streptococcae* and *Staphylococcae*	Zilberstein et al. 2007 [19]
16S rRNA sequencing		*Proteobacteria*, *Firumicutes*, *Bacteroidetes*, *Actinobacteria* and *Fusobacteria* (*Streptococcae* and *Prevotella)*	Li et al. 2009 [20] Bik et al. 2006 [21]
Molecular biology	Mice models	Increase in *Clostridia*, *Bacteroides/Prevotella* spp., *Eubacterium* spp., *Ruminococcus* spp., *Streptococci and Escherichia coli* Decrease in *Lactobacilli*	Aebischer et al. 2006 [24]
16S rRNA microarray	Chronically infected patient	Decrease of *Actinobacteriae*, *Bacteroidetes* and *Firmicutes* Increase of *Spirochetes*, *acidobacteriae* and *Proteobacteriae*	Moldonado-Contreras et al. 2011 [10]
Culture method	Under-therapies patients	Decrease of *Bifidobacteria* or *Clostridia*. Increase of *Enterococci*, *Enterobacteria* and *Peptostreptococci*	Adamson et al. 2000 [25]
16S rRNA sequencing	Under-therapies patients	Increase of *Bacteroidetes* Decrease *Actinobacteria*, *Bifidobacteriales* No modification of *Lactobacillus*	Gotoda et al. 2018 [26]
16S rRNA sequencing	Gastritis	Decrease of *Proteobacteria* (phyla) and of *Prevotella* (level) Increase of *Firmicutes* (phyla) and of *Streptococcus* (genera)	Li et al. 2009 [20]
16S rRNA microarray	Invasive gastric cancer	Decrease in *Porphyromonas*, *Neisseria* and *Streptococcus sinensis* Increase in *Lactobacillus L. coleohomonis* and *Lachnospiraceae*	Aviles-Jimenez et al. 2014 [27]
